# Supplementary material for: Uncertainty in tuberculosis clinical decision-making: An umbrella review with systematic methods and thematic analysis
Source: PLOS Glob Public Health. 2024 Jul 23;4(7):e0003429. doi: 10.1371/journal.pgph.0003429 (PMC11265660; doi:10.1371/journal.pgph.0003429)
Supplement: S1 Appendix — The search strategy was refined and tested in the MEDLINE database, and then adapted to the other databases. To restrict the search to capture SRs, while simultaneously minimizing the capture of non-SR publications, search terms and MeSH specific to SR study designs (e.g. ‘SR’, ‘qualitative evidence synthesis’) were included. A search of grey literature was not conducted. Details on the search strategy across all databases are presented in S1 Appendix. (DOCX) [file pgph.0003429.s001.docx]

**S1 Appendix. Search strategy (July 2023).**

Search strategy in Ovid-MEDLINE.

| **DOMAINS** | **TERMS** | |
| --- | --- | --- |
|  | **MeSH^1^** | **Keywords** |
| **1. Tuberculosis** | **1a** | **1b** |
|  | Tuberculosis OR  Tuberculosis, Pulmonary | Tuberculosis.mp. |
| **2. Clinical decision-making** | **2a** | **2b** |
|  | Tuberculosis/di [Diagnosis] OR Tuberculosis, Pulmonary/di [Diagnosis]OR Clinical Decision-Making OR  Health Knowledge, Attitudes, Practice OR  Practice Patterns, Physicians OR  Algorithms OR  Qualitative Research | decision-making.mp. OR  diagnostic process*.mp. OR  ((clinical or diagnostic) and algorithm*).mp.OR  prediction model*.mp. OR  (decision* adj2 treat*).mp. OR  ((clinical or risk or diagnostic or decision) and score*).mp. OR  practice pattern*.mp. OR  ((choice or decision) behavior*).mp. |
| **3. Reviews** | **3a** | **3b** |
|  | Systematic Review | review*.tw. OR  qualitative adj2 synthesis.tw. |
| Summation: (1a OR 1b) AND (2a OR 2b) AND (3a OR 3b) | | |
| Limit to year="2007 -Current^2^ " | | |

tw: title or abstract, mp: multiple places. ^1^MeSH terms were used only in those databases where available. In databases where MeSH or equivalent were not allowed, all terms were searched as keywords. ^2^Search was conducted in July 2022 and updated on 21^st^ July 2023.

| **DATABASE: CINAHL** | |
| --- | --- |
| # | search^1^ |
| S1 | Tuberculosis |
| S2 | decision making OR decision-making OR decision making process OR decision-making process |
| S3 | diagnostic process* |
| S4 | clinical algorithm* |
| S5 | diagnostic algorithm* |
| S6 | clinical score* |
| S7 | risk score* |
| S8 | diagnostic score* |
| S9 | MH diagnosis or diagnosing or diagnostics or assessment or screening |
| S10 | MH practice patterns |
| S11 | choice behav* |
| S12 | decision behav* |
| S13 | MH attitude of health personnel |
| S14 | Provider* experience* |
| S15 | Prediction model* |
| S16 | Decision* W3 treat* |
| S17 | MH qualitative research or qualitative study or qualitative methods or interview |
| S18 | S2 OR S3 OR S4 OR S5 OR S6 OR S7 OR S8 OR S9 OR S10 OR S11 OR S12 OR S13 OR S14 OR S15 OR S16 OR S17 |
| S19 | MH review of literature or literature review or meta-analysis or systematic review |
| S20 | qualitative W2 synthesis |
| S21 | S19 OR S20 OR S21 |
| S22 | S1 AND S18 AND S22 |
| S23 | Limiters- Published Date:  20070101-20231231 |

^1^ Unqualified search (Title, abstract, subject headings) unless otherwise specified

**DATABASE: EMBASE**

1 exp Tuberculosis/di [Diagnosis]

2 exp Tuberculosis, Pulmonary/di [Diagnosis]

3 decision-making.mp.

4 diagnostic process*.mp.

5 clinical algorithm*.mp.

6 diagnostic algorithm*.mp.

7 clinical score*.mp.

8 risk score*.mp.

9 diagnostic score*.mp.

10 decision score*.mp.

11 practice pattern*.mp.

12 choice behavior*.mp.

13 decision behavior*.mp.

14 choice behaviour*.mp.

15 decision behaviour*.mp.

16 provider* experience*.mp.

17 exp "Attitude of Health Personnel"/ or exp Health Knowledge, Attitudes, Practice/

18 exp Clinical Decision-Making/ or exp Decision Making/

19 exp Practice Patterns, Physicians'/

20 exp Algorithms/

21 exp Qualitative Research/

22 prediction model*.mp.

23 (decision* adj2 treat*).mp.

24 1 or 2 or 3 or 4 or 5 or 6 or 7 or 8 or 9 or 10 or 11 or 12 or 13 or 14 or 15 or 16 or 17 or 18 or 19 or 20 or 21 or 22 or 23

25 review*.ti. or review*.ab. or (qualitative adj2 synthesis.ti.) or (qualitative adj2 synthesis).ab.

26 exp "Systematic Review"/

27 25 or 26

28 tuberculosis.tw.

29 24 and 27 and 28

30 limit 29 to ("remove medline records" and "remove preprint records" and yr="2007 -Current"^1^)

^1^ 21^st^ July 2023.

| **DATABASE: SCOPUS** | |
| --- | --- |
| **#** | search |
| 1 | TITLE-ABS-KEY ( tuberculosis ) |
| 2 | TITLE-ABS-KEY ( ( diagnostic  OR  clinical  OR  decision  OR  risk  OR  prediction )  AND  ( algorithm*  OR  process*   OR  score*  ) ) |
| 3 | TITLE-ABS-KEY ( decision-making ) |
| 4 | TITLE-ABS-KEY ( practice  AND pattern* ) |
| 5 | (prediction AND model*) |
| 6 | TITLE-ABS-KEY ( (decision OR choice)  AND behav* ) |
| 7 | TITLE-ABS-KEY ( ( clinical  OR  diagnostic  OR  medical )  reasoning* ) |
| 8 | TITLE-ABS-KEY ( ( provider  OR  clinician*  OR  physician* )  AND  ( experience*  OR  knowledge  OR  decision*  OR  attitude* ) ) |
| 9 | TITLE-ABS-KEY ( decision  w2  treat* ) ) |
| 10 | 2 OR 3 OR 4 OR 5 OR 6 OR 7 OR 8 OR 9 |
| 11 | 1 AND 9 AND  PUBYEAR  >  2006 AND  ( LIMIT-TO ( DOCTYPE ,  "re" ) ) |

**DATABASE: EPISTEMONIKOS**

1 Decision-making TI, AB

2 (diagnostic OR clinical OR decision OR risk OR prediction) AND (factor* OR characteristic* OR attribute* OR algorithm* OR process* OR model* OR score* OR behav*) TI, AB

3 choice behav* TI, AB

4 practice pattern* TI, AB

5 (provider OR clinician* OR physician*) AND (experience* OR knowledge OR decision* OR attitude*) TI, AB

6 (decision to treat) OR (treatment decision) TI, ABS

7 1 OR 2 OR 3 OR 4 OR 5 OR 6

8 Tuberculosis TI, ABS

9 7 AND 8

[Filters: protocol=no, classification=systematic-review, min_year=2007, max_year=2023]

**DATABASE: COCHRANE**

SEARCH: ‘tuberculosis’ (TI, AB, KW), publication date Jan 2007-Jul 2023, in Reviews


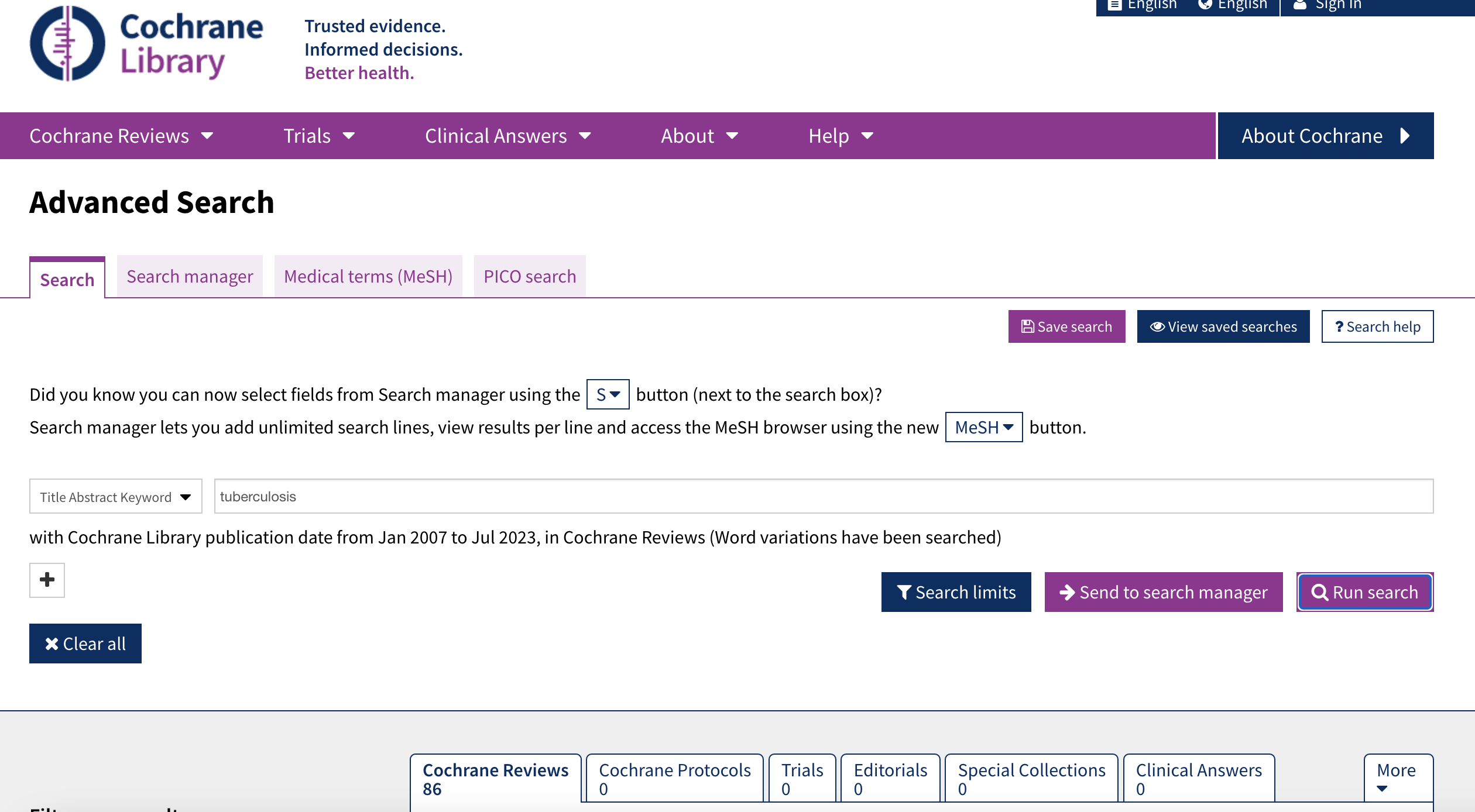


**DATABASE: PROSPERO REGISTER**

SEARCH: ‘tuberculosis’ (TI, KW), published reviews, publication date 01/06/2011-21/07/2023

**
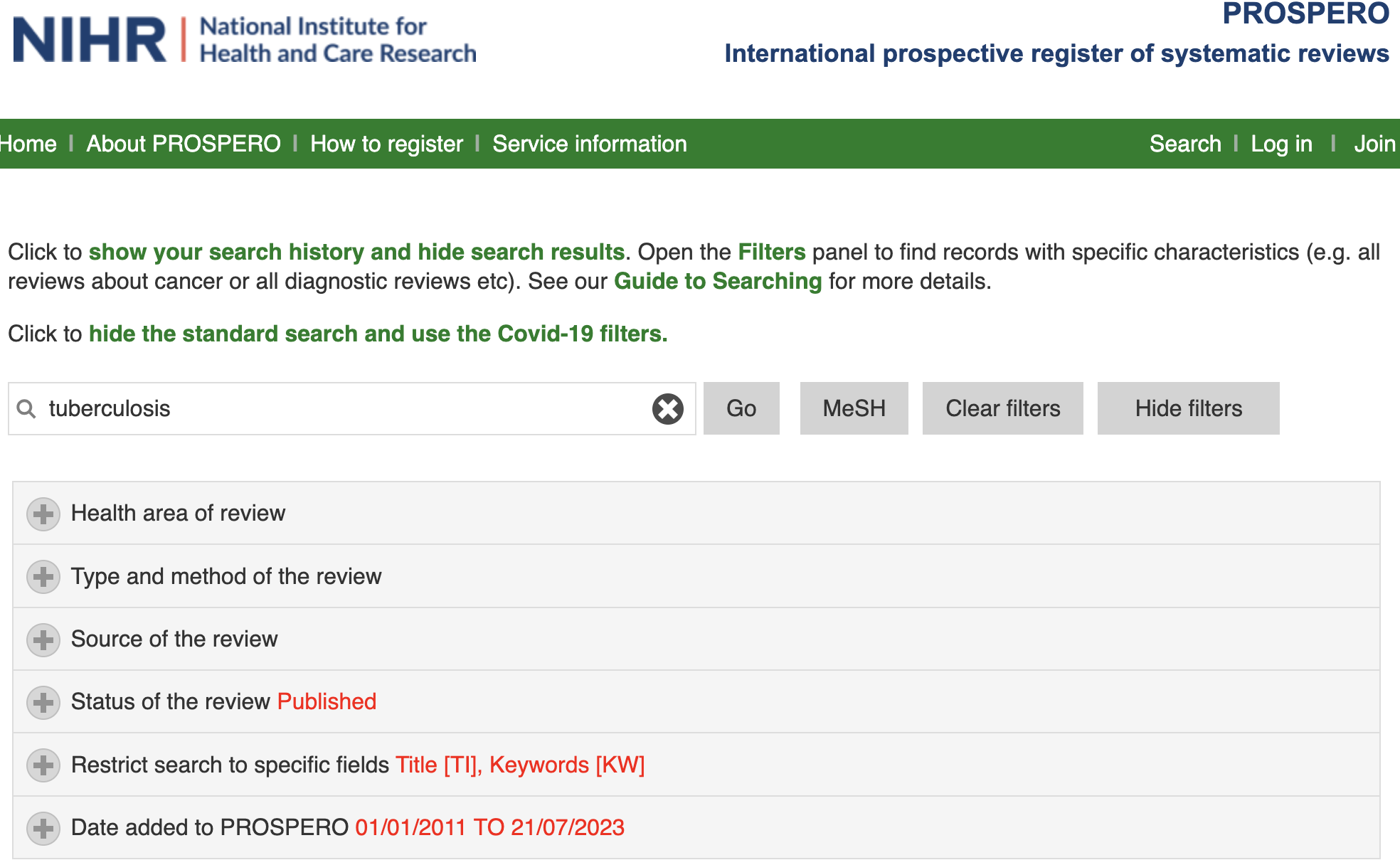
**
