# Supplementary material for: Uncertainty in tuberculosis clinical decision-making: An umbrella review with systematic methods and thematic analysis
Source: PLOS Glob Public Health. 2024 Jul 23;4(7):e0003429. doi: 10.1371/journal.pgph.0003429 (PMC11265660; doi:10.1371/journal.pgph.0003429)
Supplement: S1 Table — SRs were selected on predefined inclusion and exclusion criteria guided by the Population, Intervention, Comparison, Outcome and Study design/setting (PICOS) framework. References were pre-emptively de-duplicated in Endnote. The selected references were imported in Covidence (https://www.covidence.org/home) and re-screened for duplicates. Full texts of all potentially eligible reviews were obtained. The full text of selected papers was then examined for inclusion in the UR, based on the predefined criteria. The reason for the exclusion of each article was documented in the software for transparency and auditing purposes. (DOCX) [file pgph.0003429.s002.docx]

**S1 Table. Eligibility criteria- PICOS framework.**

|  | **Inclusion Criteria** | **Exclusion Criteria** |
| --- | --- | --- |
| **Population** | Individuals of any age with presumptive pulmonary TB (defined as clinical/pre-test suspicion or post-test suspicion despite a negative test).  Providers involved in TB diagnosis and treatment. | Non-human subjects.  Participants taking anti-TB drugs at the time of their evaluation.  Participants under investigation for drug resistant TB (DR-TB), extrapulmonary TB. |
| **Intervention** | Any finding relevant to the clinical decision-making process, including: a) use/impact of clinical or risk scores, b) use/impact of diagnostic tests, c) diagnostic algorithms, d) diagnostic thresholds, e) multi-level factors associated with TB diagnosis or treatment initiation, f) experiences of healthcare provision. | Interventions or findings focused on LTBI, DR-TB (including tests for DR-TB detection), or other diseases. |
| **Comparison** | Any if present (e.g., SRs of controlled trials) | None. |
| **Outcome** | Diagnosis of TB disease (clinical and/or microbiological reference standards) or TB treatment initiation  Changes in provider knowledge, attitude, practice in the diagnostic process (e.g., change in pre-test/post-test TB disease probability) | TB treatment adherence or treatment outcome, diagnostic test accuracy. |
| **Setting** | WHO TB and TB/HIV high burden countries. [22] | Not a WHO TB and TB/HIV high burden country. |
| **Study design** | SRs, systematic scoping reviews, meta-analyses, qualitative evidence syntheses. | Reviews without systematic methods.  Any type of primary study. |
