# Supplementary material for: Uncertainty in tuberculosis clinical decision-making: An umbrella review with systematic methods and thematic analysis
Source: PLOS Glob Public Health. 2024 Jul 23;4(7):e0003429. doi: 10.1371/journal.pgph.0003429 (PMC11265660; doi:10.1371/journal.pgph.0003429)
Supplement: S2 Table — JBI Critical Appraisal Checklist for Systematic Reviews and Research Syntheses. (DOCX) [file pgph.0003429.s003.docx]

**S3 Table. Critical appraisal of methodological quality of included reviews.**

| **Study**  (Author, year) | **Is the review question clearly and explicitly stated?** | **Were the inclusion criteria appropriate for the review question?** | **Was the search strategy appropriate?** | **Were the sources and resources used to search for studies adequate?** | **Were the criteria for appraising studies appropriate?** | **Was critical appraisal conducted by two or more reviewers independently?** | **Were there methods used to minimize errors in data extraction?** | **Were the methods used to combine studies appropriate?** | **Was the likelihood of publication bias assessed?** | **Were recommendations for policy/practice supported by the reported data?** | **Were the specific directives for new research appropriate?** |
| --- | --- | --- | --- | --- | --- | --- | --- | --- | --- | --- | --- |
| Agizew 2019 | Yes | Yes | Yes | No | Yes | Yes | Yes | Yes | No | No | Yes |
| Amare 2023 | Yes | Yes | Yes | Yes | Yes | Yes | Yes | Yes | No | Yes | Yes |
| Barnabishvili 2016 | Yes | Yes | Yes | Yes | Yes | No | No | Yes | No | No | No |
| Bell 2011 | Yes | Yes | Yes | Yes | No | Yes | No | Yes | No | No | Yes |
| Bello 2019 | Yes | Yes | No | No | No | No | Unclear | Yes | No | No | Yes |
| Bhatnagar 2019 | No | Yes | Yes | Yes | Yes | No | No | Yes | No | No | No |
| Braham 2018 | No | Yes | Yes | Yes | Yes | Yes | Unclear | Yes | No | No | Yes |
| Cai 2015 | Yes | Yes | Yes | No | Yes | Yes | Yes | Yes | No | Yes | Yes |
| DiTanna 2019 | Yes | Yes | Yes | Yes | Yes | Yes | Yes | Yes | Yes | Yes | Yes |
| Dlangalala 2021 | No | Yes | Yes | Yes | Yes | Yes | No | Yes | No | No | No |
| Engel 2022 | Yes | Yes | Yes | Yes | Yes | Yes | Yes | Yes | No | Yes | Yes |
| Getnet 2017 | Yes | Yes | Yes | Yes | Yes | Yes | No | Yes | No | No | Yes |
| Haraka 2021 | Yes | Yes | Yes | Yes | Yes | Yes | Yes | Yes | Yes | Yes | Yes |
| Krishnan 2014 | Yes | Yes | Yes | Yes | Yes | Yes | No | Yes | No | Yes | Yes |
| Lee 2022 | Yes | Yes | Yes | Yes | Yes | Yes | Yes | Yes | Yes | Yes | Yes |
| Li 2013 | Yes | Yes | Yes | Yes | Yes | Yes | No | Yes | No | Yes | Yes |
| Nathavitharana 2021 | Yes | Yes | Yes | Yes | Yes | Yes | Yes | Yes | Yes | Yes | Yes |
| Oga-Omenka 2021 | Yes | Yes | Yes | Yes | Yes | Yes | Yes | Yes | No | Yes | Yes |
| Satyanarayana 2015 | Yes | Yes | Yes | Yes | No | Yes | No | Yes | No | No | Yes |
| Shah 2022 | Yes | Yes | Yes | No | No | Yes | No | Yes | No | Yes | Yes |
| Sreeramareddy 2014 | Yes | Yes | Yes | Yes | Yes | Yes | No | No | No | No | No |
| Storla 2008 | No | Yes | Yes | Yes | No | No | No | Yes | No | No | No |
| Sullivan 2017 | No | No | Yes | Yes | No | No | Yes | Yes | No | No | No |
| Teo 2021 | Yes | Yes | Yes | Yes | Yes | Yes | Yes | Yes | No | Yes | Yes |
| Thapa 2021 | Yes | Yes | Yes | Yes | Yes | Yes | Yes | Yes | No | No | Yes |
| Yang 2014 | Yes | Yes | Yes | Yes | Yes | Yes | No | No | No | No | Yes |
| Yasobant 2021 | No | Yes | Yes | No | No | Yes | Yes | No | No | No | No |

JBI Critical Appraisal Checklist for Systematic Reviews and Research Syntheses.
