# Supplementary material for: Uncertainty in tuberculosis clinical decision-making: An umbrella review with systematic methods and thematic analysis
Source: PLOS Glob Public Health. 2024 Jul 23;4(7):e0003429. doi: 10.1371/journal.pgph.0003429 (PMC11265660; doi:10.1371/journal.pgph.0003429)
Supplement: S1 Fig — CCA was interpreted in banded thresholds: values below 5% indicated slight citations overlap, between 6–10% indicated moderate overlap, between 11–15% indicated high overlap and values above 15% indicated very high overlap. (DOCX) [file pgph.0003429.s004.docx]

**S4 Fig. Heatmap showing pairwise calculation of the CCA.**

**
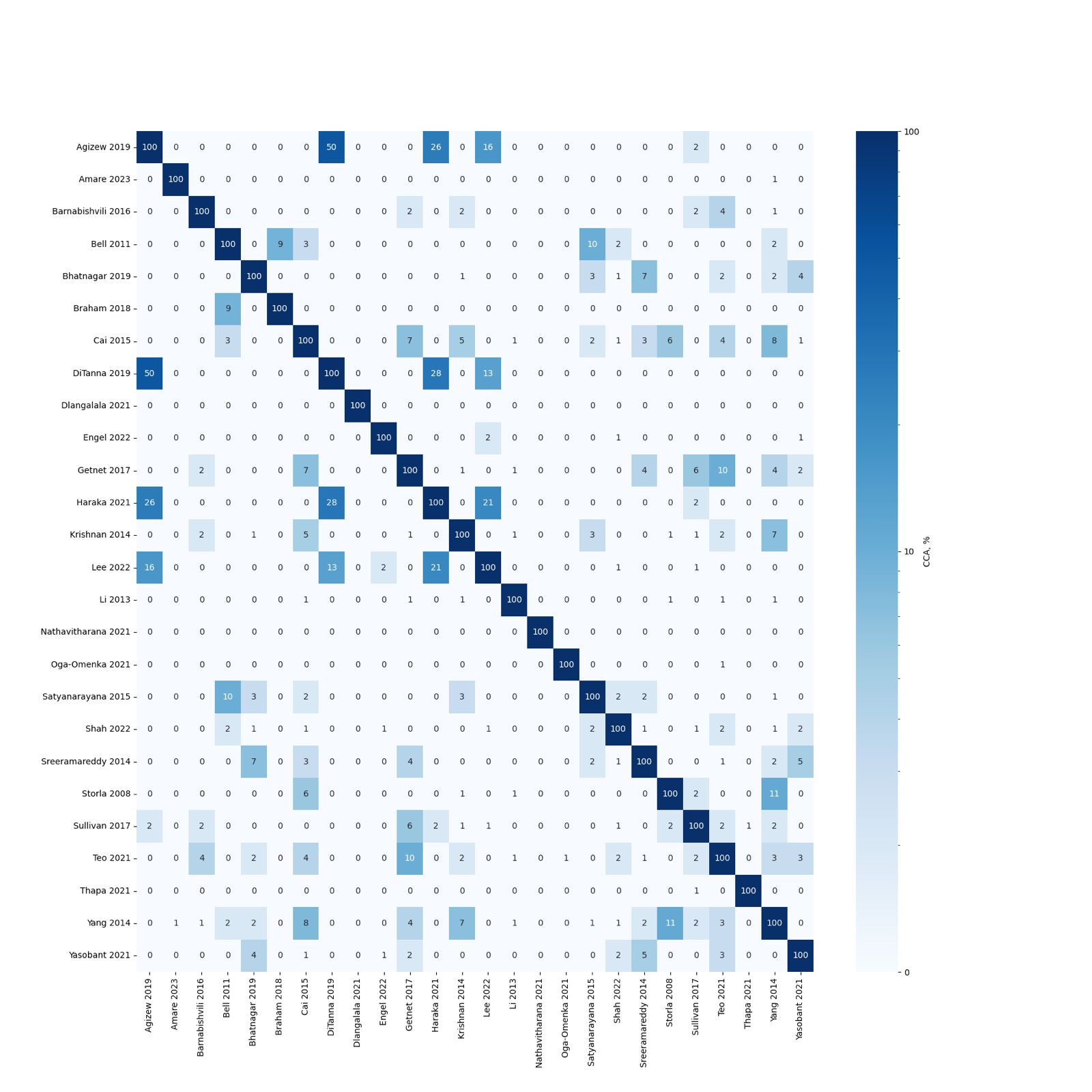
**

CCA was interpreted in banded thresholds: values below 5% indicated slight citations overlap, between 6–10% indicated moderate overlap, between 11–15% indicated high overlap and values above 15% indicated very high overlap.
