# Supplementary material for: Uncertainty in tuberculosis clinical decision-making: An umbrella review with systematic methods and thematic analysis
Source: PLOS Glob Public Health. 2024 Jul 23;4(7):e0003429. doi: 10.1371/journal.pgph.0003429 (PMC11265660; doi:10.1371/journal.pgph.0003429)
Supplement: Attachment — Submitted filename: PlosGPH reviewers responses-eb.docx [file pgph.0003429.s007.docx]

Reviewer's Responses to Questions

**Comments to the Author**

5. Review Comments to the Author

Please use the space provided to explain your answers to the questions above. You may also include additional comments for the author, including concerns about dual publication, research ethics, or publication ethics. (Please upload your review as an attachment if it exceeds 20,000 characters)

Reviewer #1: Major comments

The availability and access to diagnostic testing and the decision to test when available are two distinct concepts although there is overlap I feel at times these two concepts are being discussed interchangeably in the manuscript, it seems the objective and review focus is more around decision to test not availability of testing therefore suggest an attempt is made to be clear about these two concepts.

Thank you for this comment. We made this important distinction clearer in the manuscript introduction and made sure the concepts of decision to use a test and availability of test are not used interchangeably in the results (lines 421-429) and discuss section (lines 528-534). However, the findings from this review reinforce the idea that in real life situations there is often an inevitable overlap, with “decision” not to test being reported as a direct and inevitable consequence of diagnostics not being available or accessible (e.g., due to lack of adequate infrastructure, trainings) to clinical practitioners. Hence why the review focuses on both the determinants (i.e., what causes decisional uncertainty) but also the broader sources (i.e., what contributes to the variability of decisional outcomes) of uncertainty in the decision-making process, such as test accessibility.

Minor comments

Lines 66 & 67: best practices are not implemented (e.g., clinicians choosing quick symptom relief with low-cost pharmaceuticals over diagnostic certainty.(7)
-formatting issue here where the parantheses not closed, but also there are many reasons why best practices are not implemented therefore, perhaps this hypothesis could be better phrased as it reads more as fact, esp. given that the focus of the review is to understand the facilitators and barriers…

The typo has been corrected and the sentence has been rephrased to clarify these are examples of reasons for best practices not implemented, but there may be other possible factors.

-perhaps challenges around conflicting test results could also be mentioned, as low sensitivity and specificity in many TB tests can lead to inconsistent results when parallel testing approaches are applied for example with LAM, or DST.

Thank you for raising this important point, which however has not emerged in our findings. This was mentioned in the discussion as it raises important research question for future studies.(lines 550-552)

-Not clear how the initial scoping review was conducted.
This has been clarified in the manuscript. (lines 115-116)

-Why was diagnostics/testing not included as a broad search strategy or domain?

Searches including “diagnostics”, ”testing” or synonyms were tested in the early stages of search piloting, however did not yield an increased sensitivity while greatly reducing the specificity of the retrieved records. The final search strategy was chosen in agreement with an LSHTM librarian to optimize sensitivity and specificity of the records.

-were studies conducted in non TB endemic settings excluded? How was TB endemic settings defined? For instance in some high income countries with low TB burden, there are key populations where Tb remains endemic would these have been included/excluded?

We focused on high TB burden countries as per WHO definition, as included in the list for 2021-2025. The word “endemic” has been corrected in the whole manuscript to avoid confusion.

-please clarify why only TB patients and providers were inclusion criteria I would have thought given the focus on decision to test that you would want to include individuals with signs and symptoms of TB, but not restricting to only TB confirmed patients. Does this mean that providers of care for non TB patients were excluded? I assume not, perhaps these inclusion exclusion criteria could be further clarified in terms of how they were applied to the studies found in the initial search.

Thank you for this comment. This has been clarified in the manuscript: the population we looked at consisted of individuals with presumptive pulmonary TB and health care providers involved in TB diagnosis and treatment. Presumption of TB was defined as clinical suspicion or pre-test suspicion or post-test suspicion despite a negative test.(lines 143-144, 203-204)

-Why was DRTB excluded? I would think this a critical area to understand facilitators and barriers around decision not to test.

Thank you for this comment, we agree that DRTB is a critical and interesting area deserving its own studies due to the peculiarities of resistance profiling/testing, and second-line treatment decisions, hence why we did not include it. (lines 474-476)

-Was all of the extraction and inclusion criteria assessed by only one reviewer? Is this not a deviation from the PRISMA guidelines?

PRISMA guidelines recommend to describe in detail the process for deciding how records retrieved by the search were considered for inclusion in the review, to enable readers to assess the potential for errors in selection. (BMJ 2021;372:n160) Our approach is therefore not a deviation. Single screening enabled an efficient use of time and resources, but we acknowledge a possible higher risk of missing relevant studies. This is commented in the manuscript’s limitations. (lines 601-602)

Reviewer #2: This is a useful summary of the literature in a complex area where the effect of a diagnostic may be influenced by many (unexpected) factors. The text generally reads well and the discussion appears reasonable.

Thank you.

There is one aspect that although mentioned I feel does not receive the attention it may merit I would like the authors to consider:
In the conclusion of the abstract it is said (line 41) “This could result in delayed or missed diagnosis and treatment opportunities.”. This is correct but the lack of efficient diagnostics can also result in inappropriate treatment i.e. over treatment of people who do not have tuberculosis. I feel this is a critical point, that better diagnostics in theory might not increase the number of people diagnosed or treated but none the less increase the number of people correctly treated. This effect is mentioned in table 1 Agizew 2019 “Use of Xpert might be associated with a decrease in empiric treatment.” and also on line 386-87 “This could reflect decisions to treat some patients empirically regardless of test results.”. Also a somewhat similar effect to that seen with improved cancer screening might be expected with more accurate diagnostics; where improvements in cancer screening may paradoxically result in worse outcomes (for the group treated not the population) as treatment is targeted at the correct (ill) population and overdiagnosis is reduced. I feel the two papers mentioned below may be relevant for this discussion:
Noé, A., Ribeiro, R. M., Anselmo, R., Maixenchs, M., Sitole, L., Munguambe, K., ... & García-Basteiro, A. L. (2017). Knowledge, attitudes and practices regarding tuberculosis care among health workers in Southern Mozambique. BMC pulmonary medicine, 17, 1-7.
Garcia-Basteiro, A. L., Hurtado, J. C., Castillo, P., Fernandes, F., Navarro, M., Lovane, L., ... & Martínez, M. J. (2019). Unmasking the hidden tuberculosis mortality burden in a large post mortem study in Maputo Central Hospital, Mozambique. European Respiratory Journal, 54(3).

Thank you for picking an important point and providing useful references for our discussion. This aspect has been extensively discussed in the manuscript and we have strengthened our discussion incorporating the 2019 Garcia-Basteiro reference (lines 520-527).
